# Effects of on-pump versus off-pump coronary artery bypass grafting on myocardial metabolism

**DOI:** 10.1097/MD.0000000000015351

**Published:** 2019-04-26

**Authors:** Hong-tao Xie, Xiao-qin Kang, Shun Zhang, Yong-cang Tian, De-jun Liu, Ben-jian Bai

**Affiliations:** Department of Cardiac Surgery, The Second Affiliated Hospital of Shaanxi University of Chinese Medicine, Xianyang, China.

**Keywords:** coronary artery bypass grafting, effects, myocardial metabolism, safety, systematic review

## Abstract

**Background:**

On-pump coronary artery bypass grafting (ON-PCABG) and off-pump coronary artery bypass grafting (OF-PCABG) greatly affect myocardial metabolism (MCMB). However, no study has systematically explored and compared the impacts of ON-PCABG and OF-PCABG on MCMB. This study will aim to explore and to compare the effects of ON-PCABG and OF-PCABG on MCMB systematically.

**Methods:**

We will conduct the comprehensive literature search from the following electronic databases from inception to the present: Cochrane Library, EMBASE, MEDILINE, CINAHL, AMED and 4 Chinese databases without language restrictions. This systematic review will only concern randomized controlled trials (RCTs) and case-control studies of ON-PCABG and OF-PCABG on MCMB. The methodological quality of each entered study will be assessed by using Cochrane risk of bias tool.

**Results:**

Primary outcomes include myocardial cellular markers, myocardial lactate, oxygen utilization, pyruvate, and intramyocardial concentrations of glucose, urea and lactate. Secondary outcome comprises of glutathione, superoxide dismutase, myeloperoxidase, and oxidative stress and any other complications post surgery.

**Conclusion:**

This study will provide a high-quality synthesis and will assess and compare the effects of ON-PCABG and OF-PCABG on MCMB based on the current relevant literature evidence.

**Dissemination and ethics:**

The results will be submitted to peer-reviewed journals for publication. This study does not require ethic approval, because it only analyzes the data from published literature.

**Systematic review registration:**

PROSPERO CRD42019125381.

## 1 Introduction

Postoperative myocardial damage has been reported in several studies in patients receiving coronary artery bypass grafting (CABG) surgery.^[1–4]^ Its rate, degree and recovery mainly depend on the preoperative cardiac status, types of surgery, myocardial protection, and also the types of CABG, including on-pump coronary artery bypass grafting (ON-PCABG) and off-pump coronary artery bypass grafting (OF-PCABG).^[5–8]^ Previous studies have reported that the portion of morbidity associated with cardiopulmonary bypass mainly contributed to the inflammatory, immune responses, and intraoperative myocardial damage.^[9–11]^

Actually, multiple mediator factors may result in tissue damage through myocardial metabolism (MCMB).^[12–16]^ Although a variety of studies have reported the effects of ON-PCABG and OF-PCABG on myocardial metabolism (MCMB),^[17–22]^ no study has systematically assessed and compared the effects of ON-PCABG with OF-PCABG on MCMB. Therefore, in this systematic review, we will assess and compare the effects of ON-PCABG and OF-PCABG CABG on MCMB.

## 2 Methods and design

### 2.1 Objective

This aims of this systematic review is to investigate and compare the effects of ON-PCABG and OF-PCABG CABG on MCMB.

### 2.2 Study registration

This systematic review protocol has been registered on PROSPERO with number of CRD42019125381. The report this study has based on the guideline of Cochrane Handbook for Systematic Reviews of Interventions and the Preferred Reporting Items for Systematic Reviews and Meta-Analysis Protocol (PRISMA-P) statement guidelines.^[23]^

### 2.3 Inclusion criteria for study selection

#### 2.3.1 Type of studies

This systematic review protocol will include randomized controlled trials (RCTs) and case-control studies of ON-PCABG and OF-PCABG CABG on MCMB. The other types of studies will be excluded, including non-clinical studies, case reports, and case series.

#### 2.3.2 Type of participants

Patients with coronary heart disease of any age underwent ON-PCABG or OF-PCABG, regardless males or females will be all considered for inclusion. However, patients with severe chronic obstructive lung disease will be excluded.

#### 2.3.3 Type of interventions

Intervention of ON-PCABG will be included in the experimental group. However, the combination of ON-PCABG with other treatments will be excluded. Control intervention can include OF-PCABG CABG alone.

#### 2.3.4 Type of outcome measurements

Primary outcomes include myocardial cellular markers, myocardial lactate, oxygen utilization, pyruvate, and intramyocardial concentrations of glucose, urea, and lactate.

Secondary outcome comprises of glutathione, superoxide dismutase, myeloperoxidase, and oxidative stress. In addition, complications post surgery will also be assessed.

### 2.4 Search methods for the identification of studies

#### 2.4.1 Electronic searches

We will search the comprehensive relevant studies from the following databases from the inception to the present: Cochrane Library, EMBASE, MEDLINE, the Cumulative Index to Nursing and Allied Health Literature (CINAHL), the Allied and Complementary Medicine Database (AMED), Chinese Biomedical Literature Database, China National Knowledge Infrastructure, VIP Information, and Wanfang Data. Any randomized controlled trials (RCTs) and case-control studies concerning the effects of ON-PCABG and OF-PCABG CABG will be fully considered for inclusion. The sample size of search strategy for Cochrane Library is presented in Table 1. Identical search strategies for other electronic databases will be built and applied.

**Table 1:**
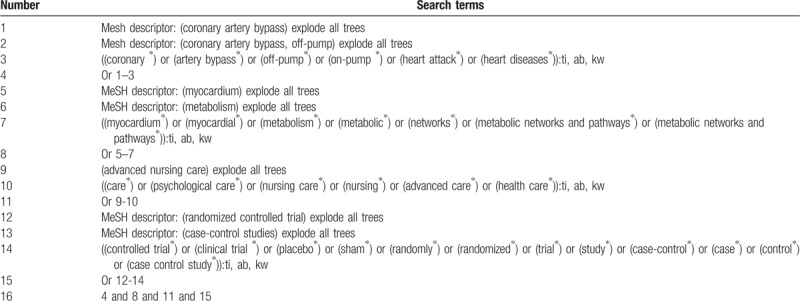
Search strategy utilized in Cochrane Library database.

#### 2.4.2 Search for other resources

Aside from the above 9 electronic databases, the sources of clinical registry and reference list of relevant studies will also be checked to avoid missing any potential eligible studies.

### 2.5 Data collection and analysis

#### 2.5.1 Study selection

**Figure 1:**
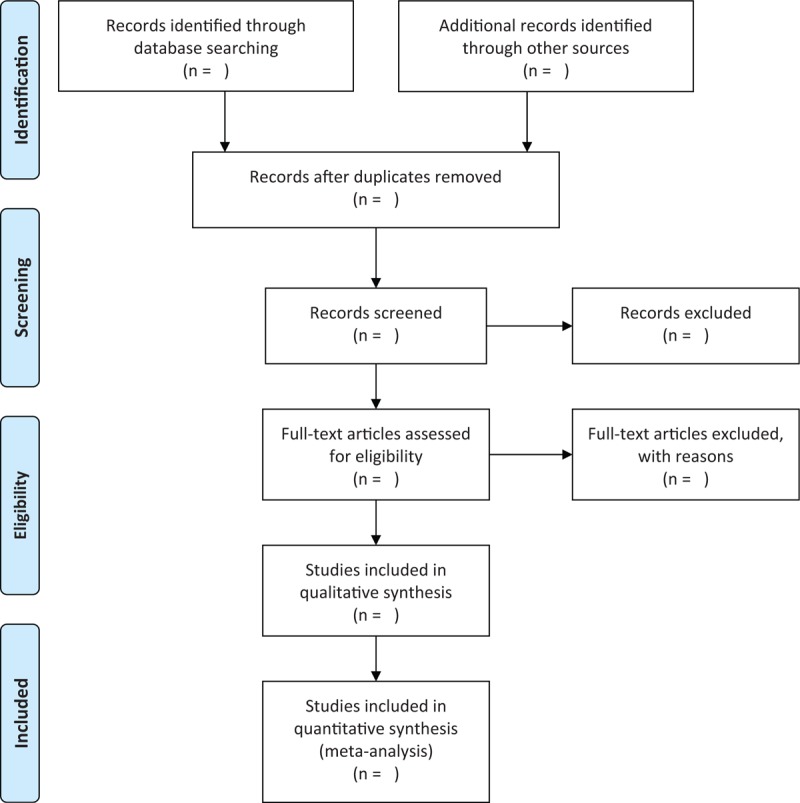
Flow diagram of study selection process.

Two review authors will independently conduct the study selection by scanning the titles and abstract summary, as well as reading the full-texts if it is necessary according to the predefined eligibility criteria. The whole procedure of the study selection is abided by the PRISMA flow chart, and it is presented in Figure 1. A third review author will be invited to solve the disagreements between the 2 review authors.

#### 2.5.2 Data extraction and management

Two review authors will independently carry out the data extraction based on the predefined data extraction sheet. It includes the following information, such as title, first author, published year, location, disease diagnosis, inclusion and exclusion criteria, sample size, age, randomization, allocation, blinding, treatment details, and outcome measurements. A third review author will be consulted if any divergences regarding the data extraction will occur between 2 review authors.

#### 2.5.3 Dealing with missing data

If any data are missing, incorrect, insufficient, or unclear, the primary authors will be contact by email to inquire those data. If we will not receive any reply, then the available data will be analyzed, and its impact will be discussed in the manuscript.

#### 2.5.4 Risk of bias assessment

We will utilize the Cochrane Handbook for Systematic Reviews of Interventions tool to assess the risk of bias for each included RCT. This tool comprises of seven domains, and we will assess each domain as high risk of bias, or unclear risk bias, or low risk of bias. Two review authors will independently assess the risk of bias for each domain in each included RCT. A third review author will resolve the disagreements by discussion if they exist between 2 review authors.

#### 2.5.5 Measurement of treatment effect

Enumeration data will be expressed as risk ratio and 95% confidence intervals, while continuous data will be described as mean difference and 95% confidence intervals. If the same outcome measured by different tools, then the data will be converted to the standardized mean difference and 95% confidence intervals.

#### 2.5.6 Assessment of heterogeneity

The heterogeneity will be checked by *I*^*2*^ test. If the value of *I*^*2*^ is less than 50%, the heterogeneity is regarded as acceptable, and a fixed-effect model will be used to pool the data. If the value of *I*^*2*^ is more than 50%, significant heterogeneity will be considered, and a random-effect will be applied to pool the data.

#### 2.5.7 Data synthesis

If the heterogeneity is acceptable, the data will be pooled by using a fixed-effect model, and a meta-analysis will be carried out by using RevMan 5.3 software. Otherwise, if the heterogeneity is substantial, the data will be pooled by using a random-effect model, and a subgroup analysis will be conducted. If the heterogeneity is acceptable (normally *I*^*2*^ ≤ 50%) after the subgroup analysis, a meta-analysis will be performed. Otherwise, the data will not be pooled and a meta-analysis will not be carried out anymore. Instead, a narrative summary will be described.

#### 2.5.8 Subgroup analysis

Subgroup analysis will be performed in accordance with the different interventions, controls, and outcome measurements.

#### 2.5.9 Sensitivity analysis

Where appropriate, sensitivity analysis will be conducted to check the robust of the pooled data by eliminating the impact of low-quality studies.

#### 2.5.10 Publication biases

Funnel plot will be carried out if sufficient studies are included (normally more than 10 studies).^[24]^ In addition, we will also conduct Egg regression for quantitative analysis.^[25]^

## 3 Discussion

This study will be performed to evaluate and compare the effects of ON-PCABG and OF-PCABG on MCMB based on the current relevant clinical literature evidence. To our best knowledge, it is the first systematic study to assess and compare the effects of ON-PCABG and OF-PCABG on MCMB. Its results will summarize latest evidence on the effects of ON-PCABG and OF-PCABG on MCMB. This evidence may be very helpful for the future researchers and clinical practice.

## Author contributions

**Conceptualization:** Hong-Tao Xie, Yong-Cang Tian, De-Jun Liu, Ben-Jian Bai.

**Data curation:** Hong-Tao Xie, Xiao-Qin Kang, Shun Zhang, De-Jun Liu, Ben-Jian Bai.

**Formal analysis:** Hong-Tao Xie, Xiao-Qin Kang, De-Jun Liu.

**Funding acquisition:** Ben-Jian Bai.

**Investigation:** Ben-Jian Bai.

**Methodology:** Hong-Tao Xie, Xiao-Qin Kang, Shun Zhang, Yong-Cang Tian, De-Jun Liu.

**Project administration:** Ben-Jian Bai.

**Resources:** Hong-Tao Xie, Xiao-Qin Kang, Shun Zhang, Yong-Cang Tian.

**Software:** Hong-Tao Xie, Xiao-Qin Kang, Shun Zhang, Yong-Cang Tian, De-Jun Liu.

**Supervision:** Ben-Jian Bai.

**Validation:** Shun Zhang.

**Visualization:** Hong-Tao Xie, Yong-Cang Tian, Ben-Jian Bai.

**Writing – original draft:** Hong-Tao Xie, Xiao-Qin Kang, Yong-Cang Tian, De-Jun Liu, Ben-Jian Bai.

**Writing – review & editing:** Hong-Tao Xie, Shun Zhang, Yong-Cang Tian, De-Jun Liu, Ben-Jian Bai.
